# A Randomized Controlled Pilot Study of the Food Order Behavioral Intervention in Prediabetes

**DOI:** 10.3390/nu15204452

**Published:** 2023-10-20

**Authors:** Alpana P. Shukla, Ampadi Karan, Katie C. Hootman, Maya Graves, Ian Steller, Brittany Abel, Ashley Giannita, Jamie Tils, Lauren Hayashi, Madlen O’Connor, Anthony J. Casper, Debra D’Angelo, Louis J. Aronne

**Affiliations:** 1 Comprehensive Weight Control Center, Division of Endocrinology, Diabetes and Metabolism, Weill Cornell Medicine, New York, NY 10021, USA; amk2028@med.cornell.edu (A.K.); brittany.abel@ucsf.edu (B.A.); asg2725@med.cornell.edu (A.G.); jamtils315@gmail.com (J.T.); anc2088@med.cornell.edu (A.J.C.); ljaronne@med.cornell.edu (L.J.A.); 2 Clinical & Translational Science Center, Weill Cornell Medicine, New York, NY 10021, USA; 3 Institute of Human Nutrition, Columbia University, New York, NY 10032, USA; vfy9002@nyp.org (M.G.); ian.steller@westernu.edu (I.S.); lauren.hayashi@ufl.edu (L.H.); madlen.oc@gmail.com (M.O.); 4 NewYork Presbyterian-Weill Cornell Medicine Psychiatry, New York, NY 10021, USA; 5 Western University of Health Sciences, Pomona, CA 91766, USA; 6 Department of Medicine, University of California San Francisco Health, San Francisco, CA 94143, USA; 7 University of Florida College of Medicine, Gainesville, FL 32610, USA; 8 Queen’s University School of Medicine, Kingston, ON K7L 3L4, Canada; 9 Department of Population Health Sciences, Weill Cornell Medicine, New York, NY 10021, USA; ded2011@med.cornell.edu

**Keywords:** diet quality, body weight, HbA1c, glucose tolerance, dietary counseling

## Abstract

(1) Background: Prior research in individuals with overweight/obesity and prediabetes or type 2 diabetes has shown that the ingestion of protein-rich food and non-starchy vegetables before concentrated carbohydrates (a carbohydrate-last food order) led to lower postprandial glucose excursions over 180 min, compared to eating the same foods in the reverse order. To expand upon this research, we sought to examine the feasibility and impact of carbohydrate-last food order behavioral intervention on glucose tolerance (GT), HbA1c, weight, and nutrient intake in adults with prediabetes in the real world over a 16-week span. (2) Methods: A total of 45 adults with overweight/obesity and prediabetes were randomized to receive 4-monthly standard nutritional counseling (C) or standard nutritional counseling plus carbohydrate-last food order counseling (FO) sessions (NCT# NCT03896360). (3) Results: The FO group decreased in body weight (−3.6 ± 5.7 lbs, *p* = 0.017), and trended toward lower HbA1c (−0.1 ± 0.2, *p* = 0.054). The C group weight trended lower (−2.6 ± 6.8 lbs, *p* = 0.102) without altering HbA1c (−0.03 ± 0.3, *p* = 0.605). GT was unchanged in both groups after 16 weeks. Changes in weight, HbA1c, and GT were similar between groups. Sensitivity analysis of pre-COVID participants showed significant weight loss in the FO group (−5.9 ± 5.3 lbs, *p* = 0.003) but not in C group (−1.0 ± 6.8 lbs, *p* = 0.608). After 16 weeks, the C group significantly reduced its daily intake of calories, fat, protein, and grains whereas the FO group increased its daily intake of vegetables and protein. There were 17 (94%) FO participants that reported high intervention adherence and 13 (72%) reported it was easy to eat protein/vegetables before carbohydrates. (4) Conclusions: A carbohydrate-last food order is a feasible behavioral strategy in individuals with prediabetes that improves diet quality, notably increasing protein and vegetable intake.

## 1. Introduction

Parallel with increasing rates of obesity, the prevalence of prediabetes in the US has grown to 38% of the adult population [1]. Although estimates vary by population characteristics and the definition of prediabetes, about 10–50% of individuals with prediabetes will progress to overt diabetes within the next 5 to 10 years [2]. Notably, overweight/obesity can double the lifetime risk of developing diabetes [3]. Accumulating evidence also points to prediabetes being a high-risk metabolic condition associated with a multitude of current and future diseases, including cardiovascular disease, non-alcoholic fatty liver disease, neuropathy, chronic kidney disease, cancer, dementia, and all-cause mortality [2,4,5].

Data from multiple intensive lifestyle intervention studies directed toward diet modification, increased physical activity, and weight loss in diverse patient populations have shown a 40–70% relative risk reduction in developing diabetes compared with standard care [6,7,8,9]. While there is evidence to support a variety of eating patterns tailored to individual preferences and metabolic goals in individuals with prediabetes [10], reducing overall calorie intake and glycemic load remains an important component of nutrition counseling. However, adherence to low-carbohydrate diets is challenging. Some of these challenges include the hedonic response to sugar triggered by the release of dopamine possibly leading to compulsive eating, as well as cultural and economic barriers [11,12]. An alternative, novel approach, researched by our group and others, to attenuating the glycemic effect of a meal is to consume concentrated carbohydrates at the end of a meal, after protein and non-starchy vegetables (“carbohydrate-last food order intervention”) [13,14,15,16]. In a previous crossover study of patients with metformin-treated type 2 diabetes (T2DM), the ingestion of protein-rich food plus non-starchy vegetables before concentrated carbohydrates reduced the incremental areas under the curve for glucose (iAUC0–180) and incremental glucose peaks by over 50%, compared to eating the same foods in the reverse order [17]. In addition, our group also found that levels of the hunger hormone ghrelin were more suppressed 3 h after a carbohydrate-last meal, suggesting this strategy may have implications for satiety and weight management [17]. Furthermore, the glycemic effects of a carbohydrate-last food order were replicated in a subsequent study in individuals with prediabetes [18]. This study additionally showed that post-meal glucose spikes were similarly reduced by ~40% when non-starchy vegetables alone or with protein were consumed prior to the carbohydrate-rich component of the meal.

This prospective pilot study expands what is known from previous research on nutrient order and tests the feasibility and metabolic effects of this behavioral intervention in the real world. A 16-week carbohydrate-last food order behavioral intervention was implemented in adults with prediabetes to assess its feasibility and impact on glucose tolerance (GT), HbA1c, weight, and nutrient intake. We postulated that a carbohydrate-last food order was a feasible behavioral strategy in individuals with prediabetes and that the addition of food order counseling to standard nutritional counseling would improve glucose tolerance, HbA1c, and nutrient intake, while reducing body weight.

## 2. Materials and Methods

### 2.1. Study Design

This was an open-label, randomized controlled pilot study. The study protocol was approved by the Weill Cornell Institutional Review Board (IRB # 1807019463) and registered on ClinicalTrials.gov (NCT# NCT03896360). Enrolled participants gave written informed consent.

### 2.2. Participants and Recruitment

The participants were recruited between February 2019 and October 2021 via an institutional research database following a review of electronic medical records, and using flyers posted within and around an academic medical center. Potential subjects were pre-screened via a standardized phone interview and all interested subjects who met the preliminary eligibility criteria underwent formal screening. English-speaking, male and female individuals 21 years of age or older who were weight-stable, defined as having <5% body weight change in the 6 months preceding the enrollment date, with a body mass index of 25–40 kg/m^2^, and that had prediabetes (HbA1c 5.7–6.4%) were eligible for participation in the study. The exclusion criteria were as follows: (1) previous diagnosis of T2DM or Hb1A1c ≥ 6.5%, fasting glucose > 125 mg/dL on screening, or 2 h glucose ≥ 200 mg/dL under an oral glucose tolerance test (OGTT); (2) the use of any oral/ injectable medications used to treat diabetes; (3) being on over-the-counter or approved/off-label weight loss medications; (4) being pregnant or breastfeeding; (5) previous bariatric surgery; (6) use of psychotropic and/or other medications known to significantly impact weight unless on a stable dose for ≥6 months; (7) an eGFR < 45 mL/min; (8) significant hepatic, cardiac, gastrointestinal, neurologic, or other medical illness that would preclude participation in the study; (9) untreated hypothyroidism or other endocrine disorders; or (10) being deemed unsuitable in the investigator’s opinion. Following the baseline assessments, participants were randomly assigned to either standard counseling (C) or standard counseling plus carbohydrate-last food order counseling (FO) using permuted block randomization. The first patient was randomized in February 2019 and the last patient completed the study in January 2022.

### 2.3. Intervention Overview

#### 2.3.1. Standard Counseling

Participants in both groups received 30 min of standard nutritional education and counseling at the baseline visit by a registered dietitian who was blinded to study assignments. The dietitian reviewed the baseline dietary intake record completed by each participant and counseled them on the tenets of a balanced, healthful diet in accordance with the USDA MyPlate framework [19]. Both groups received 15 min of standard nutritional counseling from research assistants at weeks 4, 8, and 12, consisting of education on portion sizes and macronutrients, healthy beverages, and limiting salt intake, respectively. The participants in both groups were advised not to alter their level of physical activity for the duration of the study.

#### 2.3.2. Food Order Counseling

**Figure 1:**
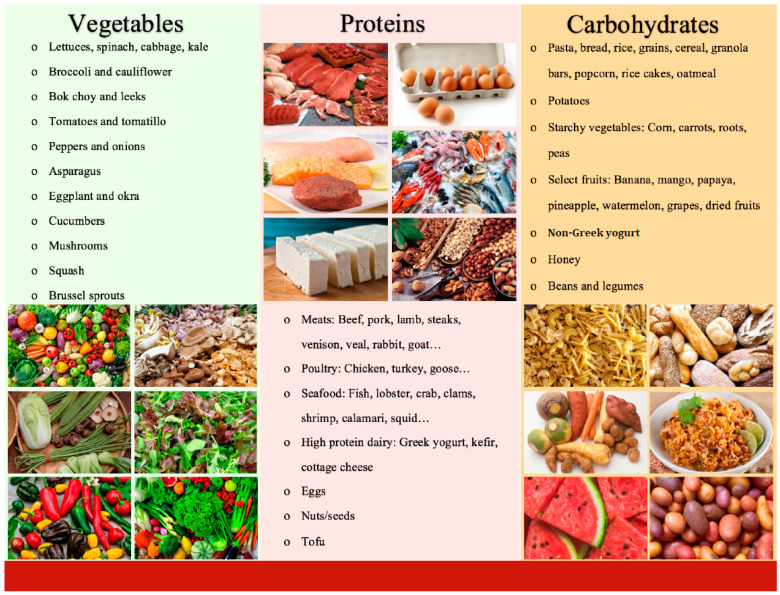
Handout given to patients categorizing foods into macronutrient groups.

Participants in the carbohydrate-last (FO) group received additional carbohydrate-last food order counseling for 15 min by research assistants at baseline and weeks 4, 8, and 12. There were three graphs illustrating the results of previous food order intervention studies shown to participants to enhance their understanding of the influence of dietary intervention on blood glucose, insulin, and ghrelin responses in acute conditions. The food order counseling initially focused on the clear categorization of foods into the three categories of interest: vegetable, protein, and carbohydrate. This required recategorization of some traditional food types with a high carbohydrate content to be moved around within the traditional food category placements. For example: peas, corn, fruit, milk, and non-Greek yogurt were placed into the carbohydrate category whereas other dairy products such as cheeses, cottage cheese, and Greek yogurt were placed into the protein category (Figure 1).

**Figure 2:**
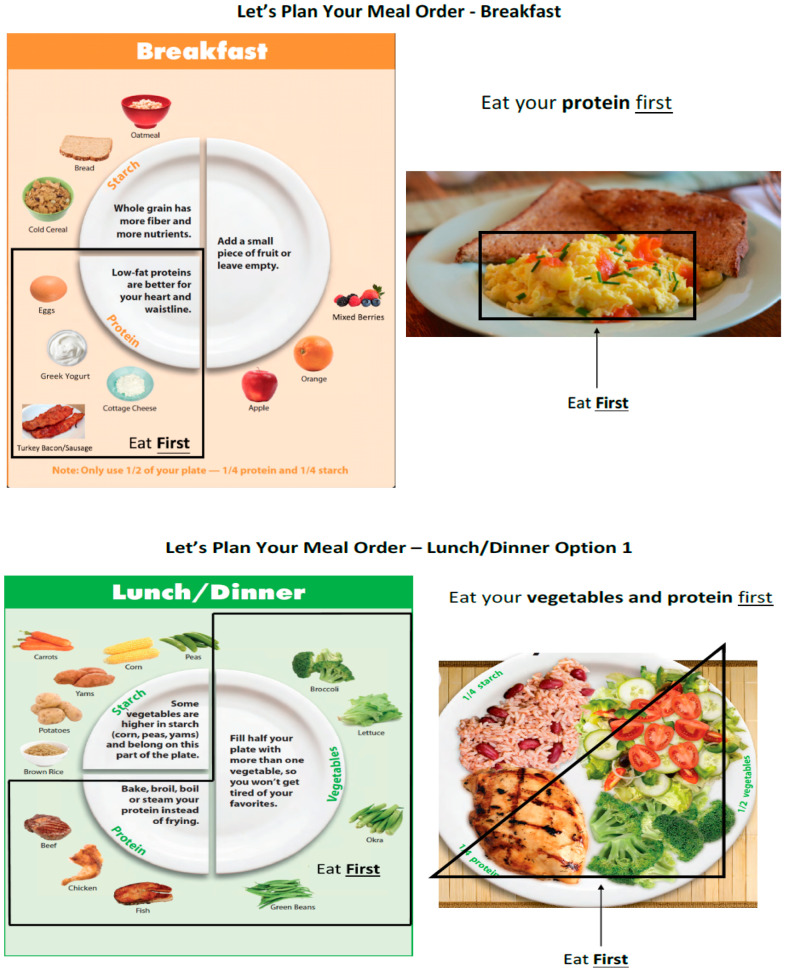

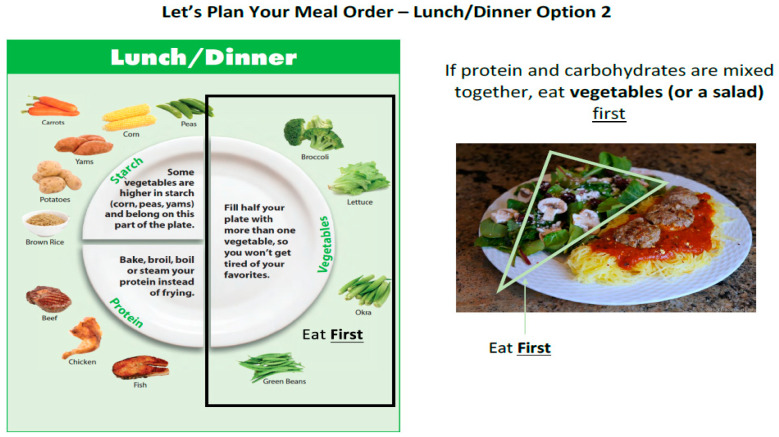
Handout given to patients outlining the carbohydrate-last food order intervention using the basic framework of the USDA MyPlate.

The second component of food order counseling focused on advising patients to consume their vegetables and protein portions before their carbohydrates at each meal. For meals where this was not feasible, such as when consuming a sandwich, patients were advised to start the meal with a salad or other non-starchy vegetables. Handouts were provided to patients outlining the carbohydrate-last food order intervention using the basic framework of USDA MyPlate and categorizing common foods into macronutrient groups (Figure 1 and Figure 2).

Additionally, the participants in the FO group were instructed to record after every meal consumed “yes” if they followed the food order for the meal and “no” if they did not follow the food order. Subjects were told to carry this out daily for the entirety of their study participation. These records, which were termed food order logs, were analyzed to determine adherence to and the feasibility of the carbohydrate-last food order intervention.

A study exit questionnaire was administered to the participants in the FO group at the end of 16 weeks to assess the overall feasibility of the intervention (Supplementary S1). Participants in both groups attended in-clinic counseling sessions until the declaration of the COVID-19 pandemic in March 2020. Thereafter, counseling sessions were delivered via telemedicine (telephone/video visits) or in clinics based on participant preference and the prevailing pandemic-related restrictions.

### 2.4. Measures

#### 2.4.1. Oral Glucose Tolerance Test

A 75 gm oral glucose tolerance test was performed at screening and at 16 weeks after an overnight 12 h fast. Blood was sampled from an indwelling venous cannula for glucose and insulin concentrations at baseline and at 30 min intervals up to 120 min. Insulin sensitivity was assessed using the Homeostatic Model Assessment for Insulin Resistance (HOMA-IR) and the Matsuda index [20], and beta cell function using a disposition index [21].

#### 2.4.2. Dietary Intake Records

All participants were instructed to complete 3-day dietary intake records which included 2 non-consecutive weekdays and 1 weekend day within a 1-week period prior to both the baseline and end-of-study visits. A registered dietitian reviewed the dietary intake records with study participants for completeness and record quality. The records were software-analyzed (Food Processor by Esha Research, version 11.6) for daily average energy intake in kilocalories, macronutrients in grams, as well as intake of food groups as defined by the USDA MyPlate framework [19,22]. Using that framework, dietary intake of the protein group of foods and the rains group of foods were quantified as ounce equivalents, and intake of the vegetables, dairy, and fruit groups of foods were quantified as cup equivalents. A quantity equivalent for each food group is outlined as follows: Grains: ½ cup cooked rice, cereal, or pasta; 1 ounce dry rice or pasta; 1 pancake, slice of bread, roll, or small tortilla; 3 cups popped popcorn. Vegetables: 1 cup raw or cooked vegetable; 2 cups raw leafy greens. Fruit: 1 cup raw or cooked fruit; ½ cup dried fruit. Dairy: 1 ½ ounce natural cheese; 2 ounces processed cheese; 1/3 cup shredded cheese; 1 cup milk or yogurt; 2 cups cottage cheese. Protein: 1 ounce of poultry, fish, or meat; 1 egg; ¼ cup cooked beans, legumes, or tofu; ½ ounce nuts or seeds; 1 tablespoon peanut butter.

#### 2.4.3. Physical Activity

Physical activity level was assessed at baseline and week 16 using the Godin Leisure-Time Exercise questionnaire [23]. The Godin Leisure-Time Physical Activity questionnaire is a short survey designed to capture the physical activity done during an individual’s spare time. This self-administered questionnaire consists of 4 items with the first 3 seeking information on the number of times one engages in strenuous, moderate, and mild exercise in periods of 15 min within a week. A weekly leisure activity Godin score is obtained by multiplying each intensity by 9, 5, and 3 metabolic equivalents, respectively, and then summed. The score obtained is then compared to categories with a Godin score of >24 units = active, 14–23 units = moderately active, and <14 units = sedentary (Supplementary S2).

#### 2.4.4. Statistical Analysis

Descriptive statistics (mean ± SD) for continuous variables and frequency (%) for categorical variables) were computed for the baseline demographic and clinical characteristics of the patient cohort, and for responses to the exit survey administered to the FO group. The number of participants in the C and FO groups with improvement in glucose tolerance defined as ≥15% reduction in 2 h glucose from baseline to week 16 were compared using Fisher’s exact test. Changes in clinical outcomes (HbA1C, weight, BMI, blood lipids, insulin sensitivity, and disposition index) and nutrient intake (calories, fat, protein, carbohydrate, fiber, grains, fruit, dairy, and vegetables) between the C and FO groups were compared using independent samples *t*-tests or Wilcoxon rank-sum tests as appropriate. Changes in the clinical and nutritional outcomes from baseline to week 16 follow-up were compared within groups using paired *t*-tests. These analyses were repeated in a subset of patients who completed the study prior to the start of the COVID-19 pandemic (13 March 2020 cut-off). All tests were two-sided and the statistical significance was evaluated at the 0.05 alpha level. Analyses were performed in SAS version 9.4 (Cary, NC, USA).

## 3. Results

A total of 80 individuals were screened, 45 were randomized (C 24, FO 21), and 39 completed the study (C 21, FO 18). In the FO group, three subjects withdrew due to the COVID-19 pandemic, and in the C group, three subjects withdrew due to personal reasons (Supplementary S3). The demographics and baseline variables including weight (191.1 lbs vs. FO 190.8 lbs), HbA1c (6% vs. 6%), and caloric intake (2131.9 kcal vs. FO 1972.2 kcal) were similar between groups, except fiber intake, which was greater in the control group (24.9 ± 10.6 g vs. FO 18.3 ± 6.4 g, *p* = 0.023). Exercise levels did not differ between groups at baseline or week 16 (Table 1).

The number of participants achieving a 15% reduction in 2 h glucose tolerance was 2 out of 14 in the FO group and 4 out of 21 in the C group (Fisher’s exact test *p* = 1.000). Within the FO group, body weight decreased from baseline to week 16 (−3.6 ± 5.7 lbs, *p* = 0.017; −1.8% ± 2.8, *p* = 0.012), and there was a trend toward improvement in HbA1c (−0.1 ±0.2, *p* = 0.054) (Table 2). The control group weight trended lower (−2.6 ± 6.8 lbs, *p* = 0.102; −1.6% ± 3.5, *p* = 0.048) without altering HbA1c (−0.03 ± 0.3, *p* = 0.605). Changes in weight, HbA1c, lipids, and glucose tolerance (GT) were not significantly different between groups. There were no significant changes in the HOMA-IR or Matsuda index in either group; however, a trend toward improvement in the oral disposition index was observed in the FO group (38.4% ± 75.6%, *p* = 0.080).

At week 16, only the C group significantly reduced its daily intake of calories (−292 ± 506 kcal/day, *p* = 0.016), fat (−13.0 ± 25.4 g/day, *p* = 0.029), protein (−15.0 ± 26.3 g/day, *p* = 0.017), and grains (−1.0 ± 1.9 ounce equivalents per day, *p* = 0.027) whereas the FO group increased its daily intake of vegetables (1.0 ± 1.6 cups per day, *p* = 0.019) and protein (2.5 ± 5.1 ounce equivalents per day, *p* = 0.050) (Table 3).

A sensitivity analysis of the pre-COVID participants showed a weight loss trend between the FO and C groups (FO −5.9 ± 5.3 lbs vs. C −1.0 ± 6.8 lbs, *p* = 0.065) (Table 2). Similar to the results from all participants, only the pre-COVID participants in the C group significantly reduced their intake of calories (−344 ± 478 kcal/day, *p* = 0.030) and fat (−18.8 ± 26.5 g/day, *p* = 0.032), while the FO group increased its intake of vegetables (1.0 ± 1.5 cups/day, *p* = 0.041 (Table 3).

The 18 participants in the food order group completed their food order log, on average, for 60.5% of their total days in the study. Participants reported following the carbohydrate-last food order intervention for 79.2% of reported meals consumed in free-living conditions over the course of 16 weeks.

At week 16, 17 FO group subjects (94%) reported high intervention adherence and 13 (72%) reported it was easy to eat vegetables/protein before carbohydrates. Although 44% felt eating vegetables/protein before carbohydrates reduced their meal enjoyment, 94% reported they were likely to continue implementing this behavioral strategy in the future (Table 4).

## 4. Discussion

In this 16-week study of adults with overweight/obesity and prediabetes spanning the COVID-19 pandemic, we demonstrated that the addition of carbohydrate-last food order counseling to standard nutrition counseling improved nutrient intake, notably increasing the intake of protein and vegetables, although reductions in body weight and HbA1c were not significantly different from those observed with standard nutrition counseling alone. Participants reported high intervention adherence and likelihood of continuing to eat protein/vegetables before concentrated carbohydrates at meals, suggesting carbohydrate-last food order is a feasible behavioral strategy.

Consuming a substantial quantity of fruits and vegetables is foundational to a healthy diet and has been shown to reduce the risk of cardiovascular disease, cancer, and all-cause mortality [24,25,26,27,28]. However, even with the known benefits of consistent vegetable intake, only 9% of adults in the United States meet the federal daily vegetable recommendation [28]. Based on our findings, food order counseling is a simple strategy that can significantly enhance vegetable consumption. Interestingly, weight loss in the FO group occurred concurrently with significant increases in vegetable and protein intake without a significant change in caloric intake, unlike in the C group, which did significantly reduce its intake of calories, fat, and protein. Dietary strategies that emphasize foods to add to the diet rather than those that focus on what to avoid are highly useful in practice, and this evidence supports the validity of the carbohydrate-last approach in adults with prediabetes.

Contrary to our postulated hypothesis, we did not observe significant differences between the study groups in glucose tolerance and HbA1c. This was not entirely unexpected given the unprecedented disruptions of the COVID-19 pandemic. No changes to physical activity were observed in either study group, supporting the objective to isolate the effects of the study intervention. Improvements in glucose tolerance have previously been described in more intensive lifestyle intervention programs that included moderate to vigorous intensity exercise [29]. Considering contextual factors, a 0.1% reduction in HbA1c within the FO group is notable. Limited data from previous prospective studies suggest that manipulating the sequence of meal ingestion improves glycemic control in individuals with T2DM [13,30].

Given previous investigations of the carbohydrate-last food order intervention demonstrate perturbations in insulin, GLP-1, and ghrelin concentrations that could potentially favor weight management, we postulated greater weight loss in the FO group. Indeed, in the pre-pandemic cohort of 24 participants (12 in each group), we did observe an average weight loss of 5.9 lbs vs. 1 lb in the FO vs. C groups. This difference attenuated over the pandemic period, when the once-monthly, in-person counseling sessions were converted into telemedicine appointments. We suspect the pre-COVID group had greater weight loss in part due to the in-person counseling visits and greater daily lifestyle stability, which may have led to greater effectiveness of FO counseling. Remarkably, while it has been shown that eating behavior changed during the COVID-19 pandemic with a shift toward increased snack frequency and preference for processed foods at the expense of fruits and vegetables [31,32], we continued to observe improved diet quality in the FO group.

Our study is novel, being the first prospective study to investigate the feasibility and effectiveness of the carbohydrate-last food order behavioral intervention in prediabetes. The study design included parallel intensity arms and the use of uniform scripts for standard counseling and structured counseling materials for CHO-last food order counseling. The implementation of this intervention is simple as it builds upon the messaging of the widely used USDA MyPlate framework, emphasizing nutrient sequencing.

Our study has limitations, including sample size and short study duration. As in most nutritional studies conducted in free living conditions, we had limited oversight of participants’ dietary intake. Dietary recall via self-reporting is commonly used for nutritional studies. However, the validity and reliability of dietary recall are limitations in nutrition trials. Additionally, adherence to carbohydrate-last food order behavior may be overestimated due to respondent bias given the nature of self-reporting [11]. A limitation of generalizability is the demographic of the cohort being primarily white, as adherence to dietary interventions can vary widely by race and ethnicity as a proxy for culture. Furthermore, a significant proportion of subjects completed this study during the COVID-19 pandemic, which likely impacted individuals in different ways, adding multiple variables, such as a change in work schedule, access to food, and a change in overall routine, that could not be accounted for.

## 5. Conclusions

In conclusion, carbohydrate-last food order is a feasible behavioral strategy that can be implemented by individuals with prediabetes. Carbohydrate-last food order behavioral counseling led to improved diet quality, notably greater vegetable and protein intake. Further research with a larger sample size and longer duration is needed to delineate its clinical impact.

## Figures and Tables

**Table 1:** Demographics and Baseline Clinical Characteristics by Intervention Group.

Outcome	Group		*p*-Values
	Food Order (N = 18)	Control (N = 21)	
	Mean (SD) or Freq. (%)	Mean (SD) or Freq. (%)	
Age (Years)	60.2 (14.4)	60.4 (10.3)	0.959
Female	12 (66.7%)	10 (47.6%)	0.232
Hispanic/Latino	0 (0%)	2 (9.5%)	0.344
Race			0.690
Asian	0 (0%)	2 (9.5%)	
Black/African American	3 (16.7%)	4 (19.1%)	
White	13 (72.2%)	14 (66.7%)	
Other	2 (11.1%)	1 (4.8%)	
Weight (pounds)	190.8 (25.9)	191.1 (31.1)	0.974
HbA1c	6.0 (0.2)	6.0 (0.2)	0.917
Calories (kcal)	1972.2 (646.6)	2131.8 (548.7)	0.409
Fat (g)	89.7 (33.2)	89.0 (28.4)	0.945
Protein (g)	94.8 (33.9)	94.2 (30.8)	0.954
Carbohydrates (g)	190.3 (83.4)	234.8 (65.9)	0.071
Dietary Fiber (g)	18.3 (6.4)	24.9 (10.6)	0.023
Food Group Intake ^b^			
Grains (oz)	5.1 (3.5)	5.9 (3.1)	0.427
Vegetables (cup)	1.9 (1.0)	2.4 (1.6)	0.281
Fruits (cup)	1.0 (1.1)	1.5 (1.1)	0.126
Dairy (cup)	1.2 (0.8)	1.6 (1.1)	0.297
Protein Foods (oz)	8.7 (4.6)	7.2 (4.3)	0.322
Physical Activity Score ^c^	36.4 (24.1)	29.5 (19.2) ^a^	0.345

All continuous variables were compared using an independent samples *t*-test, except for Food Group Intake – Fruits, which was compared using the Wilcoxon Rank Sum Test. All categorical variables were compared using Fisher’s exact test, except for sex, which was compared using the chi-squared test. ^a^ N = 19. ^b^ Food group amount shown in cups or ounces (oz) reflects cup-equivalents and ounce-equivalents, respectively. One quantity equivalent for each food group is outlined as follows: **Grains**: ½ cup cooked rice, cereal, or pasta; 1 ounce dry rice or pasta; 1 small tortilla, pancake, or roll; 3 cups popped popcorn. **Vegetables**: 1 cup raw or cooked vegetable, 2 cups raw leafy greens. **Fruit**: 1 cup raw or cooked fruit, ½ cup dried fruit. **Dairy**: 1 ½ ounce natural cheese, 2 ounces processed cheese, 1/3 cup shredded cheese, 1 cup milk or yogurt, 2 cups cottage cheese. **Protein**: 1 ounce of poultry, seafood, or meat; 1 egg, ¼ cup cooked beans, legumes, or tofu; ½ ounce nuts or seeds, 1 tbsp peanut butter. ^c^ Godin Leisure-Time Exercise Questionnaire.

**Table 2:** Comparison of changes in clinical outcomes (baselineweek 16).

	All Participants					Pre-COVID Participants Only				
Outcome	Group		*p*-Values			Group		*p*-Values		
	Food Order (N = 18)	Control (N = 21)				Food Order (N = 12)	Control (N = 12)			
	Mean (SD) or Freq. (%)	Mean (SD) or Freq. (%)	Food Order vs. Control	Within Food Order	Within Control	Mean (SD) or Freq. (%)	Mean (SD) or Freq. (%)	Food Order vs. Control	Within Food Order	Within Control
Weight (lbs.)	−3.6 (5.7)	−2.6 (6.8)	0.625	0.017	0.102	−5.9 (5.3)	−1.0 (6.8)	0.065	0.003	0.608
Weight (%)	−1.8 (2.8)	−1.6 (3.5)	0.839	0.012	0.048	−3.0 (2.5)	−0.9 (3.4)	0.097	0.001	0.366
Total Cholesterol (mg/dL)	6.1 (28.0) ^a^	−1.7 (24.2)	0.364	0.382	0.756	2.3 (29.4) ^a^	−0.4 (22.0)	0.805	0.803	0.949
Total Cholesterol (%)	5.0 (16.9) ^a^	0.8 (14.2)	0.410	0.237	0.790	3.7 (18.3) ^a^	1.0 (14.6)	0.691	0.513	0.822
HDL (mg/dL)	2.9 (13.5)	1.6 (7.2)	0.713	0.375	0.327	5.6 (14.3)	2.0 (6.8)	0.446	0.205	0.331
HDL (%)	9.6 (29.6)	4.6 (16.5)	0.532	0.186	0.211	16.4 (31.1)	7.1 (16.3)	0.369	0.095	0.160
LDL (mg/dL)	1.1 (25.7) ^b^	−0.5 (15.9) ^c^	0.823	0.863	0.898	−8.3 (23.9) ^b^	−3.7 (17.2)	0.603	0.301	0.475
LDL (%)	3.0 (23.8) ^b^	1.8 (16.6) ^c^	0.867	0.623	0.638	−3.8 (24.5) ^b^	−2.2 (16.9)	0.864	0.639	0.658
Triglycerides (mg/dL)	−4.9 (41.2) ^b^	−2.3 (28.5) ^c^	0.823	0.639	0.734	−5.5 (18.7) ^b^	5.6 (24.8)	0.258	0.376	0.452
Triglycerides (%)	−1.0 (31.2) ^b^	0.3 (28.0) ^c^	0.894	0.896	0.963	−5.7 (22.5) ^b^	8.4 (27.2)	0.205	0.442	0.308
HbA1c	−0.1 (0.2)	−0.03 (0.3)	0.364	0.054	0.605	−0.1 (0.2)	0.03 (0.3)	0.176	0.091	0.720
HbA1c (%)	−1.8 (3.8)	−0.5 (4.9)	0.363	0.056	0.627	−2.1 (3.9)	0.6 (5.3)	0.177	0.091	0.722
2−Hour Glucose (mg/dL)	2.5 (27.5) ^d^	1.3 (31.7)	0.911	0.739	0.849	−0.7 (25.0) ^a^	3.9 (39.9)	0.744	0.925	0.740
2−Hour Glucose (%)	4.0 (22.7) ^d^	0.9 (20.8)	0.681	0.522	0.844	1.5 (20.2) ^a^	2.8 (25.3)	0.891	0.814	0.709
Glucose AUC 0-120 (mg/dL)	−1073.4 (4236.0) ^b^	−200.7 (2819.5)	0.457	0.327	0.748	−178.6 (2429.7) ^a^	120.0 (3205.8)	0.805	0.812	0.899
Glucose AUC (%)	−5.9 (25.5) ^b^	−1.4 (14.8)	0.535	0.367	0.661	−0.3 (15.4) ^a^	0.2 (16.4)	0.940	0.942	0.974
HOMA-IR	0.3 (1.4) ^b^	0.5 (2.4)	0.746	0.396	0.341	0.1 (1.0) ^a^	1.2 (2.2)	0.119	0.804	0.080
HOMA-IR (%)	18.2 (58.9) ^b^	26.0 (60.6)	0.695	0.237	0.063	10.7 (27.6) ^a^	43.4 (61.6)	0.117	0.226	0.033
Matsuda Index	−0.2 (1.2) ^d^	−0.2 (1.0) ^e^	0.975	0.531	0.312	−0.03 (0.9) ^a^	−0.4 (1.0)	0.384	0.906	0.213
Matsuda Index (%)	2.3 (32.4) ^d^	−4.1 (25.8) ^e^	0.523	0.792	0.484	5.9 (28.4) ^a^	−9.4 (22.7)	0.169	0.510	0.181
Insulinogenic Index	0.2 (1.1) ^d^	0.2 (0.7) ^e^	0.985	0.424	0.136	0.4 (1.0) ^a^	0.3 (0.6)	0.744	0.242	0.140
Insulinogenic Index (%)	49.5 (106.0) ^d^	−1.0 (135.0) ^e^	0.251	0.104	0.974	37.8 (82.3) ^a^	20.8 (50.4)	0.552	0.159	0.181
Disposition Index	0.9 (3.0) ^d^	0.7 (2.5) ^e^	0.825	0.277	0.227	1.1 (3.3) ^a^	0.4 (1.8)	0.567	0.316	0.455
Disposition Index (%)	38.4 (75.6) ^d^	−3.9 (142.7) ^e^	0.272	0.080	0.905	44.4 (81.6) ^a^	12.5 (60.1)	0.295	0.101	0.486
Godin Score	−2.7 (15.6)	−1.0 (19.1) ^c^	0.766	0.469	0.822	−3.8 (16.8)	3.3 (22.3)	0.386	0.445	0.620
Godin Score (%)	25.8 (98.2)	11.8 (110.8) ^a^	0.695	0.280	0.666	34.6 (118.7)	37.5 (128.1) ^a^	0.956	0.334	0.355

^a^ N = 17; ^b^ N = 16; ^c^ N = 19; ^d^ N = 14; ^e^ N = 20.

**Table 3:** Comparison of nutrient components (week 16–baseline).

	All Participants					Pre-COVID Participants Only				
Outcome	Group		*p*-Values			Group		*p*-Values		
	Food Order (N = 18)	Control (N = 21)				Food Order (N = 12)	Control (N = 12)			
	Mean (SD) or Freq. (%)	Mean (SD) or Freq. (%)	Food Order vs. Control	Within Food Order	Within Control	Mean (SD) or Freq. (%)	Mean (SD) or Freq. (%)	Food Order vs. Control	Within Food Order	Within Control
Calories (kcal)	−64.8 (593.8)	−292.2 (505.9)	0.205	0.649	0.016	−62.4 (516.5)	−344.2 (478.3)	0.180	0.684	0.030
Fat (g)	−0.6 (36.8)	−13.0 (25.4)	0.219	0.950	0.029	12.5 (29.8)	−18.8 (26.5)	0.013	0.173	0.032
Protein (g)	10.0 (34.3)	−15.0 (26.3)	0.014	0.232	0.017	8.5 (37.8)	−16.5 (28.7)	0.082	0.452	0.072
Carbohydrates (g)	−25.1 (81.4)	−29.7 (76.7)	0.856	0.208	0.091	−45.2 (56.8)	−35.4 (73.2)	0.717	0.019	0.122
Dietary Fiber (g)	0.5 (8.3)	−1.6 (10.2)	0.474	0.786	0.469	0.5 (6.1)	−4.3 (8.8)	0.134	0.794	0.115
Grain Intake (oz)	−1.1 (4.1)	−1.0 (1.9)	0.927	0.275	0.027	−1.8 (2.8)	−1.1 (2.2)	0.469	0.045	0.123
Vegetable Intake (cup)	1.0 (1.6)	0.1 (2.0)	0.156	0.019	0.777	1.0 (1.5)	−0.4 (1.4)	0.024	0.041	0.301
Fruit Intake (cup)	−0.1 (1.3)	0.2 (1.1)	0.523	0.804	0.480	−0.3 (1.5)	0.4 (1.2)	0.257	0.521	0.319
Dairy Intake (cup)	−0.2 (0.7)	−0.4 (0.9)	0.456	0.257	0.051	−0.2 (0.7)	−0.5 (1.1)	0.326	0.467	0.116
Protein Intake (oz)	2.5 (5.1)	−0.6 (3.8)	0.035	0.050	0.482	2.5 (5.3)	−0.1 (3.7)	0.258	0.138	0.944

All food order vs. control comparisons were performed using the independent samples *t*-test, and all within-group comparisons were performed using the paired *t*-test.

**Table 4:** Exit survey descriptive statistics (n = 18; food order only).

Question	Freq. (%)
Q1 Approximately how often did you eat vegetables/protein before carbohydrates in the past 2 weeks?	
Half the time	1 (5.6)
More than half the time	11 (61.1)
Nearly all the time	6 (33.3)
Q2 Rate the following statement: It is easy to eat vegetables before carbohydrates?	
Strongly Disagree	0 (0)
Disagree	2 (11.1)
Neutral	3 (16.7)
Agree	10 (55.6)
Strongly Agree	3 (16.7)
Q3 How easy or difficult is it to eat vegetables/protein before carbohydrates for breakfast?	
Very Difficult	4 (22.2)
Difficult	3 (16.7)
Neutral	1 (5.6)
Easy	4 (22.2)
Very Easy	6 (33.3)
Q4 How easy or difficult is it to eat vegetables/protein before carbohydrates for lunch?	
Very Difficult	0 (0)
Difficult	3 (16.7)
Neutral	5 (27.8)
Easy	4 (22.2)
Very Easy	6 (33.3)
Q5 How easy or difficult is it to eat vegetables/protein before carbohydrates for dinner?	
Very Difficult	0 (0)
Difficult	4 (22.2)
Neutral	3 (16.7)
Easy	6 (33.3)
Very Easy	5 (27.8)
Q6 How easy or difficult is it to eat vegetables/protein before carbohydrates when eating at home during weekdays?	
Very Difficult	0 (0)
Difficult	1 (5.6)
Neutral	7 (38.9)
Easy	3 (16.7)
Very Easy	7 (38.9)
Q7 How easy or difficult is it to eat vegetables/protein before carbohydrates when eating at home during the weekends?	
Very Difficult	0 (0)
Difficult	6 (33.3)
Neutral	2 (11.1)
Easy	4 (22.2)
Very Easy	6 (33.3)
Q8 How often do you eat out? (i.e. take-out, restaurants, at work, at social events)	
Never	3 (16.7)
Few times a week	12 (66.7)
Daily	2 (11.1)
More than once daily	1 (5.6)
Q9 How easy or difficult is it to eat vegetables/protein before carbohydrates when eating out? ^a^	
Very Difficult	1 (5.9)
Difficult	5 (29.4)
Neutral	6 (35.3)
Easy	4 (23.5)
Very Easy	1 (5.9)
Q10 Which of these best describes your meal experience? Eating vegetables/protein before carbohydrates:	
Reduces my meal enjoyment	8 (44.4)
Does not affect my enjoyment	9 (50.0)
Increases my meal enjoyment	1 (5.6)
Q11 How likely are you to continue eating protein/vegetables before concentrated carbohydrates at meals? (N = 17 responses)	
Unsure	1 (5.9)
Somewhat likely to continue	2 (11.8)
Very likely to continue	11 (64.7)
Certainly will continue	3 (17.7)
Q12 How would you rate the frequency of the study visits? ^a^	
Not Sure	1 (5.9)
Too Little	2 (11.8)
Just Right	14 (82.4)

^a^ N = 17 respondents.

## Data Availability

The dataset pertaining to the current study is available from the corresponding author in accordance with the appropriate data use agreements and Institutional Review Board approvals for secondary analyses.

## Supplementary Materials

The following supporting information can be downloaded at https://www.mdpi.com/article/10.3390/nu15204452/s1, Supplementary S1: Study exit questionnaire. Supplementary S2: Godin Leisure-Time Exercise Questionnaire. Supplementary S3: Participant Flowchart.
