# Supplementary material for: A Randomized Controlled Pilot Study of the Food Order Behavioral Intervention in Prediabetes
Source: Nutrients. 2023 Oct 20;15(20):4452. doi: 10.3390/nu15204452 (PMC10610476; doi:10.3390/nu15204452)
Supplement: Supplementary file 1 [file nutrients-15-04452-s001.zip › nutrients-2637359-supplementary.pdf]

Subject ID:
Date:

1. **Approximately how often did you eat vegetables/protein before carbohydrates in the past 2 weeks?**

   Rarely | Less than half the time | Half the time | More than half the time | Nearly all the time

2. **Please rate how strongly you agree or disagree with the following statement: 'It is easy to eat vegetables/protein before carbohydrates.**

   Strongly Disagree | Disagree | Neutral | Agree | Strongly Agree

3. **How easy or difficult is it to eat vegetables/protein before carbohydrates for each meal?** (1 = Very Difficult, 2 = Difficult, 3 = Neutral, 4 = Easy, 5 = Very Easy)

   _____Breakfast______Lunch_____Dinner

4. **How easy or difficult is it to eat vegetables/protein before carbohydrates when eating at home during weekdays?** (1 = Very Difficult, 2 = Difficult, 3 = Neutral, 4 = Easy, 5 = Very Easy)

5. **How easy or difficult is it to eat vegetables/protein before carbohydrates when eating at home during the weekends?** (1 = Very Difficult, 2 = Difficult, 3 = Neutral, 4 = Easy, 5 = Very Easy)

6. **How often do you eat out? (i.e. take-out, restaurants, at work, and at social events)**

   Never | Few times a week | Daily | More than once daily

7. **How easy or difficult is it to eat vegetables/protein before carbohydrates when eating out?** (1 = Very Difficult, 2 = Difficult, 3 = Neutral, 4 = Easy, 5 = Very Easy)

8. **Which of these best describes your meal experience?**

   Eating vegetables/protein before carbohydrates:

   Reduces my meal enjoyment | Does not affect my enjoyment | Increases my meal enjoyment

9. **How likely are you to continue eating protein/vegetables before concentrated carbohydrates at meals?** (1 = Not at all likely to continue, 2 = Somewhat likely to continue, 3 = Unsure, 4 = Very likely to continue, 5 = Certainly will continue)

10. **Over the last 4 months, what made it difficult to eat protein/vegetables first at meals?**

11. **Over the past 4 months, what has helped you make the food order behavior change?**

12. **How would you rate the frequency of the study visits?**

    Too Much   Just Right   Too Little   Not Sure

13. **Please feel free to share any comments or concerns regarding your experience:**

Supplementary 1: Study exit questionnaire

GODIN LEISURE-TIME EXERCISE QUESTIONNAIRE

Subject #: ____________ Date: _____/ _____/_____

1. During a typical **7-Day period** (a week), how many times on average do you do the following kinds of exercise for **more than 15 minutes** during your free time (write on each line the appropriate number).

	**Times Per Week**
**A. STRENUOUS EXERCISE (HEART BEATS RAPIDLY)** (e.g., running, jogging, hockey, football, soccer, squash, basketball, cross country, skiing, judo, roller skating, vigorous swimming, vigorous long distance bicycling)	______________
**B. MODERATE EXERCISE (NOT EXHAUSTING)** (e.g., fast walking, baseball, tennis, easy-bicycling, volleyball, badminton, easy swimming, alpine skiing, popular and folk dancing)	______________
**C. MILD EXERCISE (MINIMAL EFFORT)** (e.g., yoga, archery, fishing from river bank, bowling, horseshoes, golf, snow-mobiling, easy walking)	______________

2. During a typical **7-Day period** (a week), in your leisure time, how often do you engage in any regular activity **long enough to work up a sweat** (heart beats rapidly)?
   - ❒ Never
   - ❒ Sometimes
   - ❒ Often

Supplementary 2: Godin Leisure-Time Exercise Questionnaire

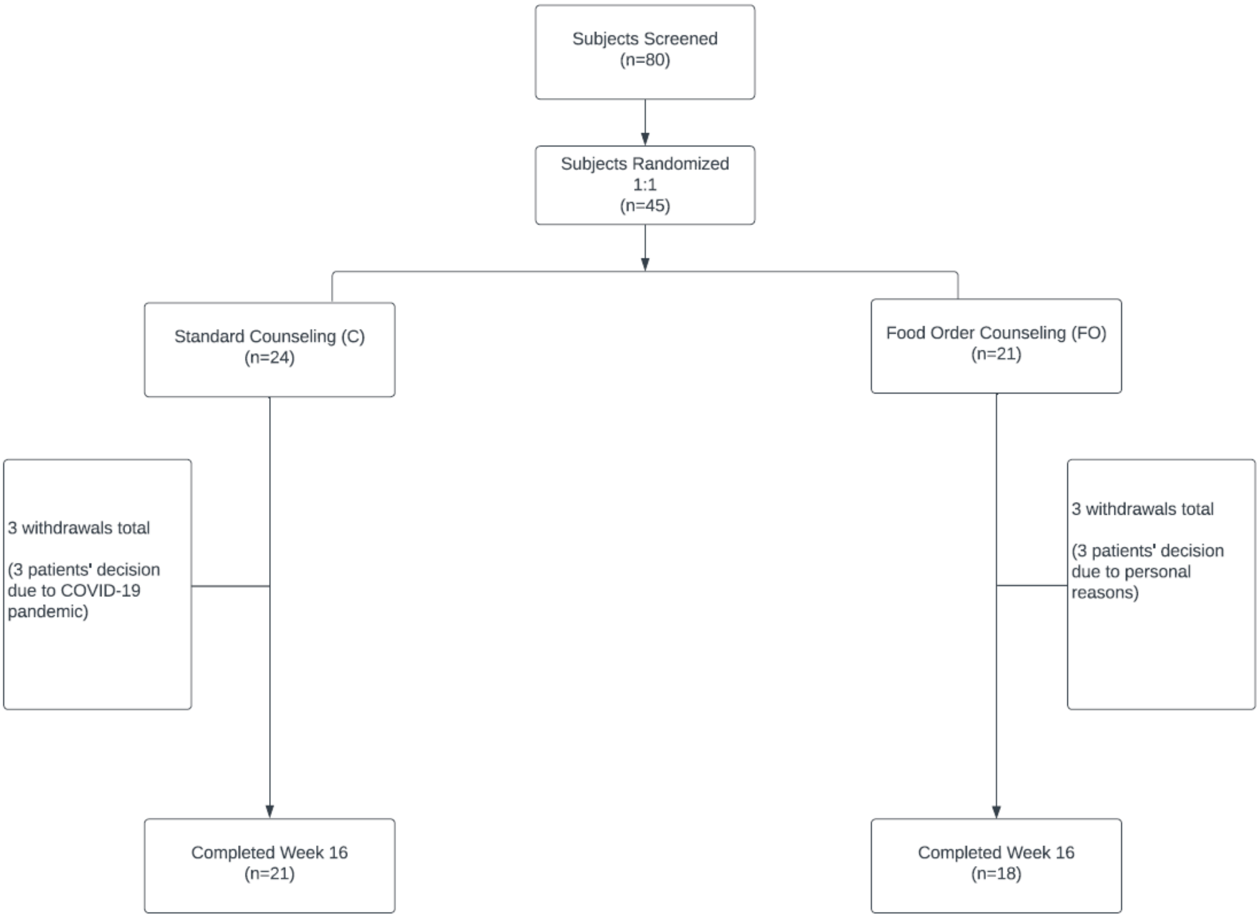

Supplementary 3: Participant Flowchart
